# Genetic polymorphisms of *ALDH2* are associated with lumbar disc herniation in a Chinese Han population

**DOI:** 10.1038/s41598-018-31491-6

**Published:** 2018-08-30

**Authors:** Qi Dong, Guoxia Ren, Kuaiqiang Zhang, Deyu Liu, Qunli Dou, Dingjun Hao

**Affiliations:** 1 Department of Spinal Surgery, Xi’an Jiaotong University Hospital Medical College Red Cross Hospital, Xi’an, Shaanxi 710054 China; 2 Department of Rehabilitation Medicine, Affiliated Hospital of Shaanxi University of Chinese Medicine, Xianyang, Shaanxi 712000 China; 3 Department of Intergrated Traditional Chinese and Western Medicine, Xi’an Chest Hospital, Xi’an, 710100 China

## Abstract

Aldehyde dehydrogenase (ALDH) is a key enzyme for the catalytic oxidation of acetaldehyde to acetic acid. Genetic polymorphisms of *ALDH2* have been associated with a wide range of diseases and cancers. However, little information is found about the association between *ALDH2* polymorphisms and lumbar disc herniation (LDH) in Chinese Han population. We investigated the association between single nucleotide polymorphisms (SNPs) in *ALDH2* and LDH risk in a case–control study that included 380 LDH cases and 692 healthy controls. Eight SNPs were selected and genotyped using the Sequenom MassARRAY platform. Odds ratios (ORs) and 95% confidence intervals (CIs) were calculated using unconditional logistic regression after adjusting for gender and age. In the allele model analysis, we found the frequency of the “A” allele of rs671 was significantly higher in LDH cases than in controls (OR = 1.414, 95%CI: 1.109–1.803, *P* = 0.005). In the genetic model analysis, we found the minor allele “A” of rs671 was associated with increased risk of LDH under log-additive model (OR = 1.42, 95%CI: 1.11–1.82, *P* = 0.0062); and the minor allele “C” of rs7296651 was associated with decreased risk of LDH under over-dominant model (OR = 0.72, 95%CI: 0.53–0.97, *P* = 0.031). Additionally, the haplotype “GGCTCACG” constructed by rs886205, rs2238152, rs4648328, rs441, rs4646778, rs671, rs11066028, and rs7296651 was associated with increased risk of LDH (OR = 1.45; 95% CI = 1.11–1.90; *P* = 0.0071). Our data shed new light on the association between genetic polymorphisms of *ALDH2* and LDH susceptibility in a Chinese Han population.

## Introduction

Lumbar disc herniation (LDH) is one of the most common diseases, which mainly caused by the varying degrees of degenerative changes of lumbar intervertebral disc^1^. LDH can stimulate or oppress the spinal nerve root and result in a series of clinical symptoms, including low-back pain, and unilateral leg pain^2^. Although it is a benign disease, it is still make patients suffer from debilitation, disability, low quality of life and work, and high costs of health care^3^. Epidemiologic studies identified that gender, age, body mass index, smoking, type of occupation and level of physical activity are primary risk factors for degeneration and herniation of lumbar disc^4^. However, subsequent twin studies showed that more than 70% of the LDH patients have genetic origin^5^, which means genetics may play a more important role than environmental factors in the occurrence and development of LDH. To date, genome wide association studies (GWAS) have identified several types of susceptibility genes associated with the degeneration of lumbar intervertebral disc, including structural related genes (*ACAN*, *COL1*, *COL9*, *FN*, *HAPLN1*, *THBS*), catabolic related genes (*MMP* and *TIMP*), inflammatory related genes (*IL1*, *IL6*, *COX2*) and so on^6^. However, this is still not enough to explain the hereditary susceptibility of LDH.

Aldehyde dehydrogenase (ALDH) is a key enzyme for the catalytic oxidation of acetaldehyde to acetic acid. Specifically, ALDH in the liver is responsible for the oxidation of ethanol (the composition of the wine) to acetaldehyde, and the acetaldehyde as a substrate is further catalyzed by ALDH and converted to non-hazardous acetic acid (the composition of the vinegar). ALDH has two isozymes, ALDH1 in the cytosol and ALDH2 in mitochondria. *ALDH2* is located on chromosome 12q24.12 region. Previous studies have demonstrated that genetic polymorphisms of *ALDH2* is associated with alcohol dependence^7^, flushing response^8^, and risk of digest tract cancers^9,10^. Recently, increased mitochondrial ALDH2 level has been found in skeletal muscle of SOD1 knockout mice, which may be involved in the nerve redox signaling in regulation of degenerative pathways in skeletal muscle^11^. However, little information is found about the association between genetic polymorphisms in *ALDH2* and the risk of LDH.

In this case-control study, we genotyped eight single nucleotide polymorphism (SNPs) in *ALDH2*: rs886205, rs2238152, rs4648328, rs441, rs4646778, rs671, rs11066028, and rs7296651, and performed a comprehensive association analysis to identify SNPs associated with LDH risk in a Chinese Han population.

## Results

A total of 380 LDH patients and 692 healthy controls were recruited in the study. The distribution of gender and age of the patient and control groups are described in Table 1. The mean age of the participants was 50.43 ± 12.27 years in the case group and 48.21 ± 10.38 years in the control group. There were no significant difference in the distributions of gender and age between LDH patients and healthy controls (*p* > 0.05). More than 90% LDH patients have herniation in L4-L5 and L5-S1. The percent of ligmaentum flayum hypertrophy and hyperplasia, dural sac compression, nerve root compression, spinal canal stenosis and abnormal MRI signal in LDH patients were 3.7%, 32.4%, 5.3%, 12.4% and 1.8%, respectively.

**Table 1:** Basic characteristic of patients with LDH and the control individuals.

Variables, No. (%)	Case (N = 380)	Control (N = 692)	*p*-value
Sex			0.248^a^
Male	228 (60.0)	390 (56.4)	
Female	152 (40.0)	302 (43.6)	
Mean age ± SD	50.43 ± 12.27	48.21 ± 10.38	0.101^b^
Degree of herniation			
Bulging	2 (0.5)		
Protrusion	348 (91.6)		
Complete prolapse	30 (7.9)		
Level of herniation			
L4-S1	148 (38.9)		
L5-S1	75 (19.7)		
L4-L5	67 (17.6)		
L3-S1	29 (7.6)		
L3-L5	20 (5.3)		
Others	41 (10.8)		
Ligamentum flavum			
Normal	366 (96.3)		
Hypertrophy	12 (3.2)		
Hyperplasia	2 (0.5)		
Dural sac			
Normal	257 (67.6)		
Compression	123 (32.4)		
Nerve root			
Normal	360 (94.7)		
Compression	20 (5.3)		
Spinal canal			
Normal	333 (87.6)		
Stenosis	47 (12.4)		
MRI signal			
Normal	373 (98.2)		
Abnormal	7 (1.8)		

^a^*P* values was calculated from Pearson’s chi-square tests.

^b^*P* values was calculated by Welch’s t tests.

All SNP call rates exceeded 99%, which was considered high enough to perform association analyses. The basic information of the *ALDH2* polymorphisms (rs886205, rs2238152, rs4648328, rs441, rs4646778, rs671, rs11066028, and rs7296651) are shown in Table 2, including gene, band, position, role, alleles and minor allele frequency (MAF). We compared the difference in frequency distributions of alleles between LDH cases and controls by Chi-square test and found one significant SNP was associated with LDH risk. The frequency of the “A” allele of rs671 was significantly higher in LDH cases than in controls (0.176 versus 0.132), which suggested that “A” allele of rs671 was a risk allele for LDH risk (OR = 1.414, 95%CI: 1.109–1.803, *P* = 0.005).

**Table 2:** Allele frequencies in cases and controls and odds ratio estimates for LDH.

SNP	Gene	Band	position	Role	Alleles A^a^/B	MAF		HWE *p*-value	OR (95%CI)	*p*-value
						Case	Control			
rs886205	*ALDH2*	12q24.12	112204427	5′UTR	A/G	0.116	0.137	0.054	0.824 (0.629–1.079)	0.159
rs2238152	*ALDH2*	12q24.12	112214459	Intron	T/G	0.279	0.284	0.023	0.976 (0.801–1.189)	0.807
rs4648328	*ALDH2*	12q24.12	112222788	Intron	T/C	0.278	0.289	0.064	0.949 (0.779–1.155)	0.602
rs441	*ALDH2*	12q24.12	112228849	Intron	C/T	0.279	0.287	0.063	0.962 (0.790–1.171)	0.698
rs4646778	*ALDH2*	12q24.12	112235783	Intron	A/C	0.279	0.288	0.077	0.956 (0.785–1.164)	0.654
rs671	*ALDH2*	12q24.12	112241766	Coding exon	A/G	0.176	0.132	0.619	1.414 (1.109–1.803)	0.005*
rs11066028	*ALDH2*	12q24.12	112245170	Intron	A/C	0.083	0.095	0.502	0.862 (0.629–1.180)	0.354
rs7296651	*ALDH2*	12q24.12	112246954	Intron	C/G	0.124	0.141	0.017	0.861 (0.661–1.120)	0.264

MAF, minor allelic frequency; HWE, Hardy-Weinberg Equilibrium; ORs, odds ratios; CI: confidence interval.

^a^Minor allele; *p value  < 0.05 indicates statistical significance.

The genotype frequencies of the *ALDH2* polymorphisms are shown in Table 3. We identified two SNPs, rs671 and rs7296651, associated with LDH risk after adjusted for gender and age. Compared with the GG genotype, the GA frequencies of rs671 polymorphism among cases were different from the controls (GA vs. GG: OR = 1.38; 95%CI = 1.04–1.84; *p* = 0.022), which suggested that the GA genotype of rs671 had an increased effect on LDH risk. Additionally, compared with individuals with the rs7296651 GG genotype, individuals with CG genotype had a decreased LDH risk (CG vs. GG: OR = 0.73; 95%CI = 0.54–0.99; *p* = 0.024).

**Table 3:** Genotypes frequencies of the SNPs and their associations with risk of LDH.

SNP	Genotypes	Genotype Frequencies		Without adjustment		With adjustment	
		Control	Case	OR (95%CI)	*P*	OR (95%CI)	*P* ^b^
rs886205	G/G	508 (73.5%)	297 (78.4%)	1	0.1	1	0.096
	A/G	176 (25.5%)	76 (20.1%)	0.74 (0.54–1.00)		0.74 (0.54–1.00)	
	A/A	7 (1%)	6 (1.6%)	1.47 (0.49–4.40)		1.58 (0.52–4.78)	
rs2238152	G/G	360 (53.1%)	200 (52.6%)	1	0.67	1	0.67
	G/T	251 (37%)	148 (39%)	1.06 (0.81–1.39)		1.06 (0.81–1.38)	
	T/T	67 (9.9%)	32 (8.4%)	0.86 (0.55–1.36)		0.86 (0.54–1.35)	
rs4648328	C/C	360 (52%)	200 (52.8%)	1	0.76	1	0.76
	T/C	264 (38.1%)	147 (38.8%)	1.00 (0.77–1.31)		1.00 (0.77–1.31)	
	T/T	68 (9.8%)	32 (8.4%)	0.85 (0.54–1.33)		0.85 (0.54–1.34)	
rs441	T/T	362 (52.3%)	199 (52.4%)	1	0.68	1	0.66
	C/T	263 (38%)	150 (39.5%)	1.04 (0.80–1.35)		1.04 (0.79–1.35)	
	C/C	67 (9.7%)	31 (8.2%)	0.84 (0.53–1.33)		0.84 (0.53–1.33)	
rs4646778	C/C	359 (52.1%)	200 (52.6%)	1	0.78	1	0.77
	C/A	263 (38.2%)	148 (39%)	1.01 (0.77–1.32)		1.01 (0.77–1.32)	
	A/A	67 (9.7%)	32 (8.4%)	0.86 (0.54–1.35)		0.85 (0.54–1.35)	
rs671	G/G	520 (75.1%)	257 (67.6%)	1	0.019*	1	0.022*
	G/A	162 (23.4%)	112 (29.5%)	**1.40 (1.05–1.86)**		**1.38 (1.04–1.84)**	
	A/A	10 (1.4%)	11 (2.9%)	2.23 (0.93–5.31)		2.26 (0.94–5.43)	
rs11066028	C/C	563 (81.6%)	321 (84.5%)	1	0.27	1	
	C/A	123 (17.8%)	55 (14.5%)	0.78 (0.55–1.11)		0.78 (0.55–1.11)	0.24
	A/A	4 (0.6%)	4 (1.1%)	1.75 (0.44–7.06)		1.90 (0.47–7.68)	
rs7296651	G/G	503 (72.7%)	294 (77.4%)	1	0.029*	1	0.024*
	C/G	183 (26.4%)	78 (20.5%)	**0.73 (0.54–0.99)**		**0.73 (0.54–0.99)**	
	C/C	6 (0.9%)	8 (2.1%)	2.28 (0.78–6.64)		2.49 (0.85–7.27)	

OR: odds ratio; 95%CI: 95% confidence interval.

^b^*P* values were calculated by unconditional logistic regression analysis with adjustments for age and gender.

^*^p <0.05 indicates statistical significance.

Next, we assumed that the major allele of each SNP was a reference allele and calculated the odd ratio and 95%CI between each variant and LDH risk under four genetic models (Table 4). Two susceptibility SNPs were found to be associated with LDH risk after the adjustment: the minor allele “A” of rs671 was associated with increased risk of LDH under log-additive model (OR = 1.42, 95%CI: 1.11–1.82, *P* = 0.0062); the minor allele “C” of rs7296651 was associated with decreased risk of LDH under over-dominant model (OR = 0.72, 95%CI: 0.53–0.97, *P* = 0.031).

**Table 4:** Association between SNPs and risk of LDH in multiple inheritance models.

SNP	Model	Genotype	Control	Case	Without adjustment				With adjustment			
					OR (95% CI)	*P*	AIC	BIC	OR (95% CI)	*P*	AIC	BIC
rs671	Dominant	G/G	520 (75.1%)	257 (67.6%)	1	0.0089*	1391.1	1401.1	1	0.011*	1383.2	1403.1
		G/A-A/A	172 (24.9%)	123 (32.4%)	1.45 (1.10–1.91)				1.43 (1.09–1.89)			
	Recessive	G/G-G/A	682 (98.5%)	369 (97.1%)	1	0.11	1395.4	1405.4	1	0.1	1387	1406.9
		A/A	10 (1.4%)	11 (2.9%)	2.03 (0.86–4.83)				2.07 (0.87–4.96)			
	Overdominant	G/G-A/A	530 (76.6%)	268 (70.5%)	1	0.03	1393.3	1403.3	1	0.038*	1385.3	1405.2
		G/A	162 (23.4%)	112 (29.5%)	1.37 (1.03–1.81)				1.35 (1.02–1.80)			
	Log-additive	—	—	—	1.42 (1.11–1.82)	0.0051*	1390.1	1400.1	1.42 (1.11–1.82)	0.0062*	1382.1	1402
rs7296651	Dominant	G/G	503 (72.7%)	294 (77.4%)	1	0.091	1395.1	1405.1	1	0.1	1387	1406.9
		C/G-C/C	189 (27.3%)	86 (22.6%)	0.78 (0.58–1.04)				0.78 (0.58–1.05)			
	Recessive	G/G-C/G	686 (99.1%)	372 (97.9%)	1	0.096	1395.2	1405.2	1	0.071	1386.3	1406.3
		C/C	6 (0.9%)	8 (2.1%)	2.46 (0.85–7.14)				2.68 (0.92–7.82)			
	Overdominant	G/G-C/C	509 (73.5%)	302 (79.5%)	1	0.029*	1393.2	1403.2	1	0.031*	1385	1404.9
		C/G	183 (26.4%)	78 (20.5%)	0.72 (0.53–0.97)				0.72 (0.53–0.97)			
	Log-additive	—	—	—	0.85 (0.65–1.12)	0.25	1396.7	1406.6	0.86 (0.66–1.13)	0.29	1388.5	1408.4

ORs, odds ratios; CI: confidence interval; AIC: Akaike’s Information criterion; BIC: Bayesian Information criterion.

^*^p value < 0.05 indicates statistical significance.

**Figure 1:**
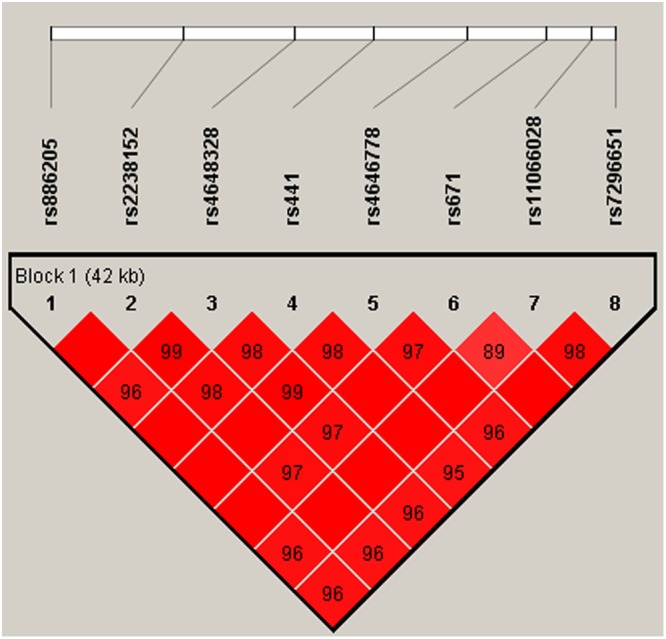
D’ linkage map for the eight SNPs in *ALDH2* The linkage disequilibrium (LD) block was constructed by rs886205, rs2238152, rs4648328, rs441, rs4646778, rs671, rs11066028, and rs7296651.

Finally, the relationship of *ALDH2* haplotypes with the risk of developing LDH was also evaluated. Figure 1 showed the linkage disequilibrium (LD) block in *ALDH2*. The association between different haplotypes and BC risk was shown in Table 5. The haplotype “GGCTCACG” constructed by rs886205, rs2238152, rs4648328, rs441, rs4646778, rs671, rs11066028, and rs7296651 was associated with increased risk of LDH after the adjustment (OR = 1.45; 95% CI = 1.11–1.90; *P* = 0.0071).

**Table 5:** Haplotype frequencies of SNPs in the *ALDH2* gene and the association with LDH risk in case and control subjects.

SNP	Haplotype	Frequency-Case	Frequency-Control	Without adjustment		With adjustment	
				OR (95% CI)	*P*	OR (95% CI)	*P*
rs886205| rs2238152| rs4648328| rs441| rs4646778| rs671| rs11066028| rs7296651	GGCTCGCG	0.416	0.44	1	—	1	—
	GTTCAGCG	0.269	0.285	1.00 (0.81–1.24)	0.98	1.00 (0.81–1.23)	1
	GGCTCACG	0.174	0.13	1.46 (1.12–1.91)	0.0058*	1.45 (1.11–1.90)	0.0071*
	AGCTCGAC	0.08	0.092	0.90 (0.64–1.27)	0.56	0.91 (0.64–1.28)	0.58
	AGCTCGCC	0.034	0.041	0.86 (0.52–1.41)	0.54	0.86 (0.52–1.42)	0.55

*p* values were calculated from two-sided Chi-squared test/Fisher’s exact test, and adjusted by gender and age.

^*^p  < 0.05 indicates statistical significance.

## Discussion

To date, several studies have investigated the association between genetic polymorphisms and risk of intervertebral disc degeneration, and identified a wide range of susceptibility genes and SNPs for degeneration of intervertebral disc in different populations^3,6^. However, due to the small sample scale and the insufficient study designs and methods in the laboratory, the results identified in previous studies need further replication, and this research direction is still worth to be further explored. The goal of this study was to assess the association between genetic polymorphisms of *ALDH2* and risk of LDH. Our findings indicate that the “A” allele of rs671 was associated with increased risk of LDH; while the “C” allele of rs7296651 was associated with decreased risk of LDH. Additionally, the haplotype “GGCTCACG” constructed by rs886205, rs2238152, rs4648328, rs441, rs4646778, rs671, rs11066028, and rs7296651 was associated with increased risk of LDH.

ALDH2 is a key enzyme for alcohol metabolism, and the *ALDH2* gene is a highly polymorphic gene. By far, previous association studies have identified more than 80 SNPs in *ALDH2* gene that associated with a wide range of disease and different types of cancers^12^. Among these SNPs, rs671 was most widely studied. Rs671, also known as Glu487Lys or Glu504Lys, is a functional SNP in exon 12 of *ALDH2*, which could destroy the combination of ALDH2 enzyme and its coenzymes and further lead to the decreased enzymatic activity of ALDH2^13,14^. Rs671 polymorphism has been found closely associated with many types of cancers, including esophageal cancer, gastric cancer, hepatocellular carcinoma, colorectal cancer, squamous cell carcinoma of head and neck, and lung cancer^12^. Beyond that, rs671 was also associated with several types of age-related disease, including late-onset Alzheimer’s disease, Parkinson’s disease, type II diabetes mellitus, coronary heart disease and myocardial infarction^15–17^. However, there is a limited number of literatures about the *ALDH2* polymorphisms associated with disease of orthopedics. In 2006, rs671 was found to be associated with osteoporosis in elderly Japanese population^18^. Until recently, one study pointed out that rs671 have a significant correlation with hip fracture^19^. Our study is the first reported that rs671 is associated with risk of LDH, which shed new light on the association between *ALDH2* polymorphisms and disease of orthopedics.

In addition to rs671, we also investigated seven SNPs in the intron region of *ALDH2*. Rs886205 polymorphism was well studied in association studies, and has been identified have association with esophageal cancer in a Chinese population^20^. Rs2238152 polymorphism and its interaction with alcohol intake was associated with risk of hypertension^21^. Rs4648328 was found to be have effect on the methadone dose and adverse reactions in drug addicts^22^. Little information is found about the rest four SNPs, rs441, rs4646778, rs11066028, and rs7296651, in literatures. In the presents study, we found that rs7296651 is associated with risk of LDH, which still needs to be confirmed in a larger Chinese cohort.

Some limitations should to be considered in our study. First, all the participants were collected from our hospital, which may only represent a fraction of Chinese population. Second, LDH is a complex disease that influenced by multiple genes and environmental factors. Because we have no data on the specific information about body mass index, smoking status, type of work and level of physical activity, we could not explore the interactions between *ALDH2* polymorphisms and environmental factors in development of LDH. Further studies will focus on the interactions between rs671 and rs7296651 in *ALDH2* and environmental factors in risk of LDH.

In conclusion, the present study provided new evidence that rs671 and rs7296651 in *ALDH2* are associated with risk of LDH in a Chinese Han population, which could be considered as potential target sites for novel treatment strategies for LDH. However, further functional study base on animal model must be conducted to demonstrate the biological role of rs671 and rs7296651 variants in the occurrence and development of LDH.

## Materials and Methods

### Subjects

All participants in our study were unrelated Han Chinese individuals and living in Shaanxi Province. A total of 380 LDH patients and 692 healthy controls were consecutively recruited between September 2015 and September 2017 at the Xi’an Jiaotong University Hospital Medical College Red Cross Hospital, Xi’an, People’s Republic of China. All the patients were recently diagnosed with LDH according to the magnetic resonance imaging (sagittal and axial images obtained with a 1.5-T imaging system), and a history of unilateral pain from the femoral or sciatic nerve to the corresponding dermatome of the nerve root for more than three months. Patients with trauma-related LDH, rheumatic, spinal tumor or spondylitis were excluded. The control group was randomly selected healthy individuals, which with no known disease, and no history of any cancers.

All subjects were at least 18 years old and were in good mental condition. All of the participants provided written informed consent.

### SNP selection and genotyping

Eight SNPs were chosen from previously published polymorphisms associated with alcohol dependence^21,23–25^, with minor allele frequencies >5% in the HapMap Chinese Han Beijing population. To date, these SNPs have not been reported for LDH susceptibility. DNA extraction and concentrations were done as previously described^26–28^. SNP genotyping was performed by the Sequenom MassARRAY RS1000, and the data analyses were completed by Sequenom Type 4.0.

### Statistical analyses

All of the statistical analyses were performed with Microsoft Excel (Microsoft Corporation, Redmond, WA, USA) and the SPSS 21.0 statistical package (SPSS, Chicago, IL, USA). Allele frequencies in the control subjects were tested for departure from Hardy–Weinberg Equilibrium (HWE) before analysis. Differences between the cases and controls in the distributions of gender, age and allele frequencies of the SNPs were evaluated using chi-square tests for categorical variables and Welch’s t tests for continuous variables. Associations between the genotypes of the *ALDH2* polymorphisms, and the risk of LDH were estimated by computing odds ratios (ORs) and 95% confidence intervals (CIs) from unconditional logistic regression analysis with adjustment for gender and age. Four models (codominant, dominant, recessive, and log-additive) were used to assess the association between each genotype and the LDH risk. Akaike’s Information criterion (AIC) and Bayesian Information criterion (BIC) were used to select the best model for each SNP. The best model was with the minimum sum of AIC and BIC. All *p* values presented in this study were two sided, and *p* = 0.05 was considered the cutoff for statistical significance.

Haploview software version 4.2 was used to analyze the association between haplotypes and the LDH. Linkage disequilibrium (LD) analysis was performed using genotype data from all the subjects. The pattern of LD was analyzed using two parameters, r^2^ and D′. Statistical significance was established when *p* < 0.05.

## Data Availability

The datasets generated during and/or analyzed during the current study are available from the corresponding author on reasonable request.
